# Follow Your Nose: A Key Clue to Understanding and Treating COVID-19

**DOI:** 10.3389/fendo.2021.747744

**Published:** 2021-11-18

**Authors:** Christopher Edwards, Oleksandra Klekot, Larisa Halugan, Yuri Korchev

**Affiliations:** ^1^ Hammersmith Hospital, Imperial College, London, United Kingdom; ^2^ Clinical Pharmacology Department, Vinnytsia National Pirogov Memorial Medical University, Vinnytsia, Ukraine; ^3^ Infection Department, Vinnytsia State Clinical Hospital #3, Vinnytsia, Ukraine

**Keywords:** COVID-19, mineralocorticoid receptor, spironolactone and dexamethasone, ATP - adenosine triphosphate, anosmia and ageusia

## Abstract

This paper suggests that ATP release induced by the SARS-CoV-2 virus plays a key role in the genesis of the major symptoms and complications of COVID-19. Infection of specific cells which contain the Angiotensin-Converting Enzyme 2 (ACE2) receptor results in a loss of protection of the Mineralocorticoid Receptor (MR). Local activation by cortisol stimulates the release of ATP initially into the basolateral compartment and then by lysosomal exocytosis from the cell surface. This then acts on adjacent cells. In the nose ATP acts as a nociceptive stimulus which results in anosmia. It is suggested that a similar paracrine mechanism is responsible for the loss of taste. In the lung ATP release from type 2 alveolar cells produces the non-productive cough by acting on purinergic receptors on adjacent neuroepithelial cells and activating, *via* the vagus, the cough reflex. Infection of endothelial cells results in the exocytosis of WeibelPalade bodies. These contain the Von Willebrand Factor responsible for micro-clotting and angiopoietin-2 which increases vascular permeability and plays a key role in the Acute Respiratory Distress Syndrome. To test this hypothesis this paper reports proof of concept studies in which MR blockade using spironolactone and low dose dexamethasone (SpiDex) was given to PCR-confirmed COVID-19 patients. In 80 patients with moderate to severe respiratory failure 40 were given SpiDex and 40 conventional treatment with high dose dexamethasone (HiDex). There was 1 death in the HiDex group and none in the SpiDex. As judged by clinical, biochemical and radiological parameters there were clear statistically significant benefits of SpiDex in comparison to HiDex. A further 20 outpatients with COVID-19 were given SpiDex. There was no control group and the aim was to demonstrate safety. No adverse effects were noted and no patient became hyperkalaemic. 90% were asymptomatic at 10 days. The very positive results suggest that blockade of the MR can produce major benefit in COVID19 patients. Further larger controlled studies of inpatients and outpatients are required not only for SARS-CoV-2 infection per se but also to determine if this treatment affects the incidence of Long COVID.

## Introduction

Early in the course of the SARS-CoV-2 pandemic it became clear that one of the commonest symptoms was loss of smell and/or taste. Self-reported alterations in smell and taste were detailed in a meta-analysis of 3563 confirmed cases of COVID-19 (1). They found that the overall prevalence of smell or taste impairment was 47% rising to 67% in patients with more severe disease. In about 20% of patients it was an isolated presenting symptom. Han et al. (2) reviewed the pathophysiology of anosmia in upper respiratory tract infections. Many rhinovirus infections of the nasal olfactory epithelium produce post-viral anosmia which persists for weeks or months until the cell damage is repaired. Post-viral anosmia has been reported with HCoV-229E infection with the olfactory dysfunction lasting more than 6 months. This corona virus does not use ACE2 to get into cells. Conductive or obstructive anosmia is often found with the common cold virus. In comparison with these infections SARS-CoV-2 produces a type of anosmia with a much more rapid recovery. Lechien et al. (3) looked at this in 417 patients with COVID-19. Of these 357 (85.6%) had olfactory dysfunction (79.6% were anosmic and 20.4% hyposmic). Olfactory dysfunction persisted after the resolution of other general symptoms in 63% cases. However, of these 72.6% recovered olfactory function within the first 8 days after resolution of the other clinical features of the disease.

**Figure 1:**
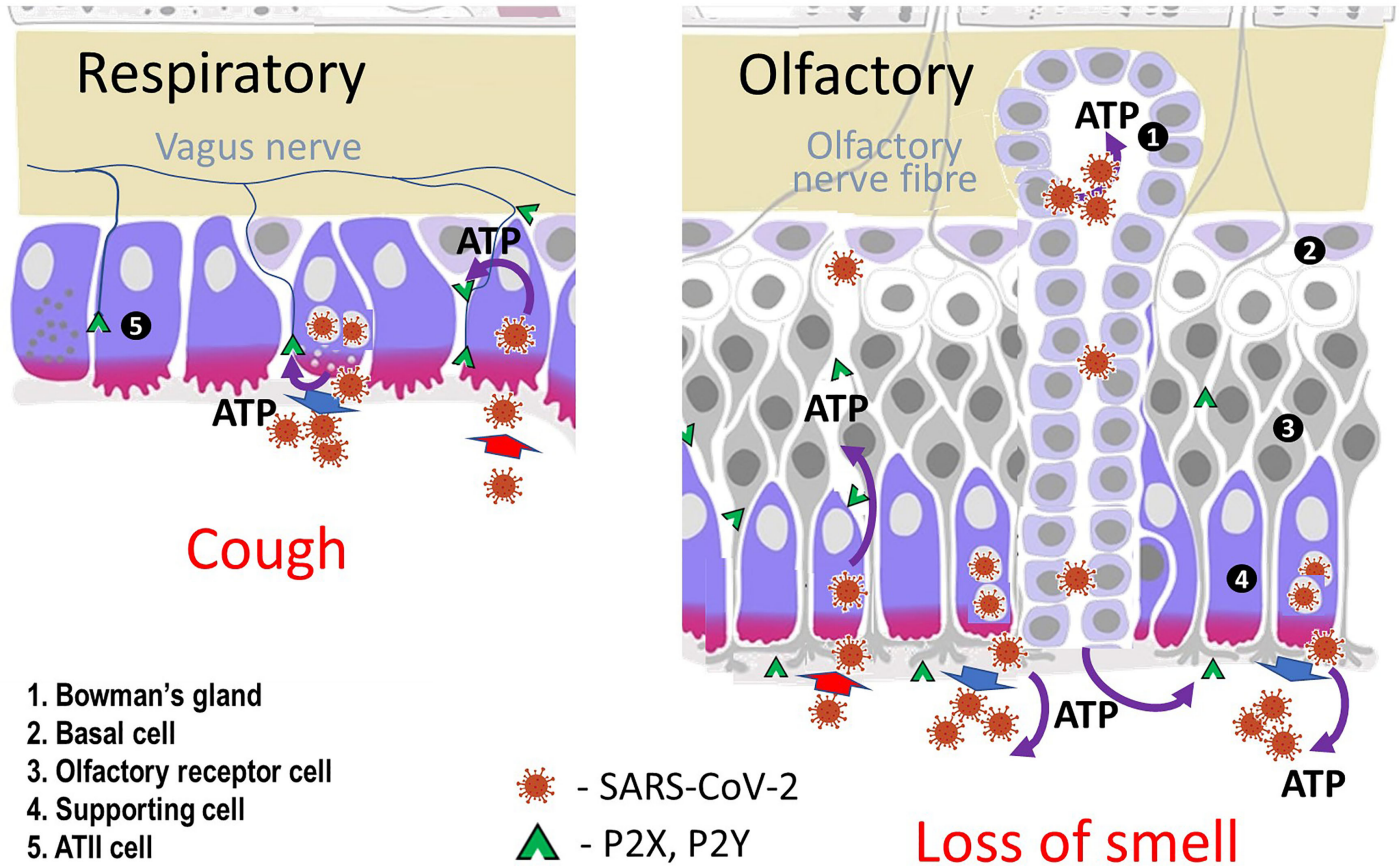
Diagram of cells in olfactory and respiratory epithelia. Sustentacular (supporting) cells, cells lining Bowman’s glands in the nose and type II alveolar cells in the lung express ACE2 receptors, Mineralocorticoid Receptors and the enzyme inactivating cortisol (11β-HSD2).

Given that the virus uses the ACE2 receptor to enter cells, the location of the receptor is likely to be of importance in relation to anosmia. Olfactory sensory neurons do not express genes for ACE2 but these are found in support (sustentacular) cells and in the cells of Bowman’s gland in both human and mice olfactory cells (**Figure 1**) (4, 5).

As with the nose ACE2 receptors are not found in the taste bud receptor cells (6). Studies in mice found that there was an enriched subpopulation of epithelial cells expressing ACE2 in the basal region of non-gustatory filiform papillae but not in the taste buds. ACE2 is expressed in human salivary glands in the cell membrane of ducts including the interlobular excretory ducts. Both mucinous and serous acini lack ACE2 (7). The squamous epithelium of the tongue was negative for ACE2.

Thus the SARS-CoV-2 virus causes a very high incidence of anosmia and ageusia, often with no clinical evidence of an inflammatory response, but without directly infecting the olfactory nerves and taste buds. In the nose this appears to be by infecting adjacent cells. In the mouth the most likely targets would seem to be non-gustatory filiform papillae and salivary glands. This paper suggests that it is the release of ATP by cells infected by the virus that is responsible. The role that this mechanism might play in the complications of COVID-19 has been previously published (8). That work focused mainly on the role that the Mineralocorticoid Receptor might play in endothelial pathology. This paper relates more specifically to epithelial cells.

After the SARS-CoV-2 virus enters the cell using the ACE2 receptor this results in a failure of ACE2 conversion of angiotensin II to angiotensin (1-7). High levels of angiotensin II stimulate NADPH oxidase and hence produce high levels of Reactive Oxygen Species (ROS).

The key cells infected by the virus in the nose, salivary glands and in the lung (type 2 alveolar cells) (**Figure 1**) contain not only ACE2 receptors but also Mineralocorticoid Receptors (MRs) and the NAD-dependent enzyme 11β-Hydroxysteroid Dehydrogenase type 2 (11β-HSD2) which converts cortisol (compound F) into inactive cortisone (compound E) (9, 10).

**Figure 2:**
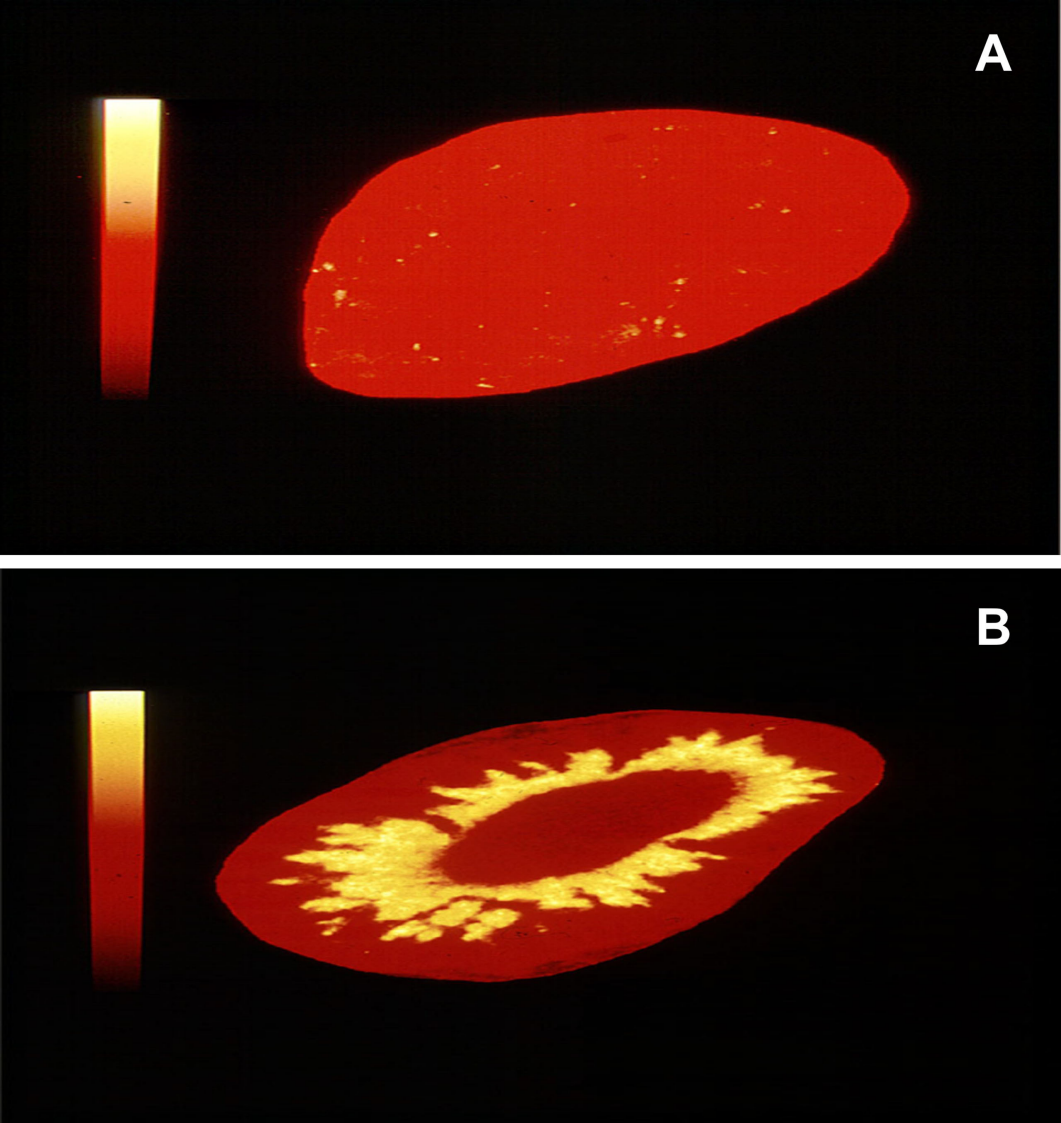
Autoradiographs of rat kidney. In **(A)** ^3^H-corticosterone was given to a rat 1 hour before death. The kidneys were removed, cryostat sectioned and exposed to Ultrafilm. In **(B)** the experiment was repeated after simultaneous administration of both ^3^H-corticosterone and glycyrrhizic acid to inhibit 11β-HSD2 [Method of Sutanto et al. (17)]. The protected MR in rat A does not bind to the labelled corticosterone (equivalent of cortisol in man). Removal of the MR protection allows the labelled steroid to bind to the MR in the renal tubules (rat B). These autoradiographs were taken as part of previously published experiments (16).

Oxidative stress produces loss of the normal MR protective mechanism resulting in cortisol activation of the receptor in man and by corticosterone in rats (8, 11–13). The consequences of the loss of 11β-HSD2 were first identified in patients with apparent mineralocorticoid excess who present with severe hypertension and hypokalaemia. This can be due to a congenital deficiency of the enzyme (14) or to inhibition of the enzyme by, for example, the active component of liquorice, glycyrrhetinic acid (GE) (15). Studies in rats confirmed the remarkable importance of this protective mechanism (16). **Figure 2** is a demonstration of this.

Using epithelial cells we have shown that the addition of aldosterone produced a rapid release of ATP from the basolateral aspects of the cells (18). Using Scanning Ion Conductance Microscopy (SICM) we found that aldosterone produced cell contraction and this was associated with the opening of the epithelial sodium channel (ENaC). The released ATP acted on basolateral purinergic receptors on the cell and adjacent cells. This opened calcium channels and the increase in intracellular calcium produced cell contraction. If we incubated the cells with hexokinase and glucose to consume ATP as it was released, the cells did not contract and ENaC did not open.

In renal epithelial cells ATP is released in a polarised manner either at the apical or basolateral surfaces for the autocrine and paracrine regulation of epithelial function. ATP has very different effects when released into the basolateral as compared to the apical compartment. Thus *in vivo* and *in vitro* experiments have shown that apical P2 receptors inhibit sodium reabsorption in collecting ducts (19). If ATP is acting in a paracrine way in the nose, for example, it would seem likely that both apical and basolateral release are important. A paper by Li et al. (20) suggests how this might be achieved.

They examined ATP-containing lysosomal exocytosis by astrocytes exposed to oxidative stress. The background to the experiments was the observed decrease in intracellular ATP when the cells were exposed to oxidative stress. They found that this stimulated the accumulation of ATP within the lysosomes. In untreated cells the lysosomes were around the nucleus. After addition of hydrogen peroxide they moved away towards the cell membrane. The higher the dose of hydrogen peroxide the greater the accumulation of ATP-containing lysosomes at the margins of the cell. They then showed that the release of ATP by exocytosis of the lysosomes was calcium dependent.

If this sequence predicts what is happening in nasal, salivary and alveolar epithelia then it is worth considering the following model.

1. The virus infects the epithelial cell using the ACE2 receptor for entry.
2. The loss of the ACE2 receptor results in angiotensin II stimulation of NADPH oxidase with consequent high levels of Reactive Oxygen Species (ROS).
3. The oxidative damage produced by ROS has a series of consequences including loss of the protection of the Mineralocorticoid Receptor (MR) and activation of ATP accumulation in lysosomes. The lysosomes move to the periphery of the cell.
4. Cortisol activates the unprotected MR which stimulates the release of ATP into the basolateral compartment of the cell. This then acts on purinergic receptors which open Ca++ channels.
5. The increase in intracellular calcium stimulates contraction of the cell and exocytosis of the ATP containing lysosomes from the apical surface of the cell with consequent high levels of extracellular ATP.

Purinergic receptors play a key role in both taste and smell (21, 22). ATP signalling has been shown to be crucial for communication from taste buds to gustatory nerves (23). Mice lacking P2X2 and P2X3 purinergic receptors exhibit no taste activity in chorda tympani or glossopharyngeal nerves when stimulated with taste stimuli from any of the 5 classical taste quality groups (21). Hegg et al. demonstrated that ATP released by the olfactory epithelium after noxious stimuli could act as a neuromodulatory substance independent of efferent innervation (22). They found that both P2Y and P2X receptor activation by exogenous and endogenous ATP significantly reduced odour responsiveness. Conversely purinergic receptor antagonists increased odour induced calcium transients. They concluded that ATP-mediated odour suppression is a novel mechanism for reducing olfactory sensitivity during exposure to olfacto-toxins and this might be a neuroprotective mechanism. It would appear that the SARS-CoV-2 virus might well activate this mechanism.

One of the authors (CRWE)has previously suggested that the mechanism described above for loss of smell and taste with COVID-19 is also responsible for the non-productive cough that is a common presentation in patients infected with the SARS-CoV-2 virus (8). The virus enters the type II alveolar cell using the ACE2 receptor, stimulates ROS production and activates the Mineralocorticoid Receptor. ATP is released from the cell and acts on adjacent neuroepithelial bodies. This stimulates the purinergic receptors on the vagal nerve and hence the cough reflex (**Figure 1**).

Elevated inflammatory cytokines play an important role in the pathogenesis of severe COVID even though the evidence for a cytokine storm is debated (24). The NLRP3 inflammasome is a key part of this (25). Patients with severe COVID have elevated levels of IL-1β and IL-6. Activation of the inflammasome requires a two-step process with first priming and then activation (26). The priming signal increases the expression of Pattern Recognition Receptors (PRR). ROS can act as a priming signal. Extracellular ATP is a danger signal that triggers macrophage NLRP3 inflammasome by activating the purinergic P2X7 receptor (26). It is thus not surprising that infection of key cells such as type II alveolar cells leads to NLRP3 inflammasome activation (25). This raises an important question. If it was possible to lower the peri-cellular ATP levels would this inhibit the release of the NLRP3 inflammasome and hence the inflammatory response?

Experiments using endothelial cells have shown that activation of the Mineralocorticoid Receptor stimulates the release of ATP, activates purinergic receptors, increases intracellular calcium and hence exocytosis of Weibel-Palade bodies (27). These contain the Von Willebrand Factor (VWF) and angiopoietin-2. These are markedly elevated in patients admitted to hospital with COVID-19 and very high levels are predictive of death. A previous paper suggested that the release of ATP by these cells is a key mechanism underlying the exocytosis of these molecules and hence the major complications of SARS-CoV-2 infection (Acute Respiratory Distress Syndrome and multiple microthrombi) (8).

A recent study has shown that the expression of ACE2 differs markedly between endothelial and epithelial cells (28). The authors compared expression of the ACE2 protein in endothelial cells (including lung microvascular and aortic endothelium) with nasal epithelial cells. The cells were treated either with live SARS-CoV-2 or a pseudo-virus for an hour and then imaged at 24hours using post-infection luminescence. When compared to the nasal epithelial cells the endothelial cells had low or undetectable levels of ACE2 and showed no susceptibility to infection with the virus. They suggested that the vascular dysfunction and thrombosis seen in severe COVID was a result of factors released by adjacent infected epithelial cells. There is a potentially very important alternative explanation.

Kaneko et al. (29) looked at ACE2 receptor mRNA and protein expression in human microvascular cells both as a monolayer and in a 3D-printed vascular model of the middle cerebral artery which enabled them to examine the effect of shear stress. ACE2 levels were low in monolayer endothelial culture as found by others (28), in marked contrast with the 3D-model where both gene expression and protein expression levels of ACE2 progressively increased with both vessel size and flow rates. SARS-CoV-2 liposomes were found in endothelial cells in the 3D model but not in monolayer culture. Further work using a human specific intra-cranial artery stenosis model showed that ACE2 expression within the stenotic portion was notably higher than in monolayer cells. They then looked at S-protein liposomes as a way of looking at endothelial susceptibility to infection with SARS-CoV-2. They found that these labelled liposomes were bound to the endothelium primarily within the stenotic portion. This confirmed other work showing the effect of shear stress on ACE2 (30).

Of particular interest was that they coated the 3D models with fibronectin and then seeded them with endothelial cells. In stable blood vessels endothelial cells rest on a basement membrane consisting mainly of laminin and type IV collagen. This changes to fibronectin in unstable cells and is a key part of vascular remodelling in vascular disease (31). Thus patients with cardiovascular disease and those with diabetes have a change in their vascular basement membrane with collagen being replaced by fibronectin (31). Shear stress in these vessels produces marked activation of the NFκB pathway. All forms of vascular remodelling are associated with increased oxidative stress. It is thus likely that the 3D stenotic model and its susceptibility to infection with spike protein liposomes does reasonably explain why patients with vascular comorbidities such as hypertension, cardiovascular disease and diabetes, in addition to age-related atherogenesis, are prone to COVID-19 complications (8).

If cortisol activation of the Mineralocorticoid Receptor (MR) is playing a key role in the pathogenesis of COVID-19 and its complications it suggests that MR blockade could be an effective therapy. In this context it is vital to appreciate that free cortisol circulates at 250 times higher levels than free aldosterone. Thus, if MR receptor blockers alone are used they have to be given at a higher than normal dose. An alternative is to combine the MR blocking drug with dexamethasone, not as an anti-inflammatory drug, but to suppress cortisol secretion. Dexamethasone unlike cortisol does not bind to the MR. Some very important evidence suggests that MR receptor blocking drugs may be very useful in both severe and mild COVID-19.

Vicenzi et al. (32) gave canrenone intravenously to COVID-19 patients admitted to hospital with moderate to severe respiratory failure. Canrenone is a metabolite of spironolactone and has the advantage that it can be given intravenously. Out of 69 consecutive patients 39 in Group A were given conventional treatment which included methylprednisolone, hydroxychloroquine, lopinavir/ritonavir, and anakinra. Thirty patients in Group B had this together with intravenous canrenone. The mortality in Group A was 37% and in Group B 13%. In Group A 47% patients required invasive ventilation. In Group B this was only 20%. Kaplan-Meier statistics showed that results both for all-cause mortality (p<0.005) and composite outcomes (p<0.0004) were highly significant.

Does MR receptor blockade have any effect if it is given earlier in the disease? The evidence here is limited but a paper by Lin et al. (33) is of particular interest. They gave what they called antiandrogen therapy to patients presenting early with COVID-19. The anti-androgens used were spironolactone and dutasteride (a 5α-reductase inhibitor). These drugs were given in conjunction with anti-viral drugs [azithromycin (AZI), hydroxychloroquine (HCQ), ivermectin (IVE) and nitazoxanide (NTZ)]. This was an outpatient treatment protocol using PCR tested nasal swabs. The patients did not have respiratory distress as judged by shortness of breath or oxygen saturation less than 92% and had a wide range of symptoms and not just fever, dry cough, anosmia or ageusia. Viral swabs were performed every 7 days and all patients completed clinical manifestation questionnaires on days 1/2/3/7/14/30/60. The primary end point was viral detection greater than 7 days and the secondary outcome the presence of symptoms greater that 7 days. 475 patients were included in the study, 223 females and 242 males. Of these 215 received spironolactone and only 28 dutasteride.

Anti-viral treatment alone in various combinations (AZI+HCQ, AZI+HCQ+IVE, AZI+IVE, AZI+NTZ) had no significant effect on lowering viral detection beyond 7 days, recovery in less than 7 days or symptoms lasting less than 7 days. Adding spironolactone (100mg twice daily until recovery) to AZI+HCQ significantly reduced viral detection beyond 7 days (p<0.011), recovery beyond 7 days (p<0.001) and symptoms greater than 7 days ((p<0.0066). The most effective combination was adding spironolactone to AZI+NTZ (all p<0.0001). Unfortunately, they did not test spironolactone alone. Dutasteride also had a significant effect when given with AZI+NTZ but only 28 patients were given this. The authors suggested that the benefits of the drugs were in relation to the role of the androgen protease TMPRSS2, the cell surface protein that primes the viral spike protein and enables viral cell entry. The dose of spironolactone used is highly likely to have produced Mineralocorticoid Receptor blockade. Unfortunately, in their multivariate logistic regression of symptoms they did not include anosmia or ageusia so the effect of treatment on this was unclear.

To determine if spironolactone and low dose dexamethasone were effective in the treatment of COVID-19 we have carried out the following studies.

## Materials and Methods

### Study 1

A series of 80 consecutive patients with PCR-confirmed COVID-19. All patients were hospitalised for radiologically confirmed pneumonia due to SARS-CoV-2 infection and were cared for in the Vinnytsia Hospital Infection Unit. All patients received usual care treatment which included: Ceftriaxone 2 g/day intravenously, Ambroxol 90 mg/day orally, Enoxaparine 0.6 ml (6000 IU subcutaneously daily).

40 patients were given dexamethasone 16 mg orally per day for 10 days (HiDex group I) and 40 given dexamethasone 4 mg per day and spironolactone 100 mg per day for 10 days (SpiDex group II). Clinical characteristics, co-morbidities and clinical parameters of the patients are listed in **Table 1**. Patient baseline characteristics and co-morbidities were similar in both groups, age range 28-84 years.

Table 1General characteristics, clinical parameters at baseline of Study 1.

**Table:** 

Characteristics	Overall (n=80)	Group I (n=40/80)	Group II (n=40/80)	Group I vs group II
General characteristics				
Sex, females (%)	52 (65)	27 (67.5)	25 (62.5)	NS
Age, years, mean±SD	61.8±11.1	62.4±9.8	61.1±12.3	NS
Hypertension, n (%)	53 (66.3)	26 (65)	27 (67.5)	NS
Ischemic heart disease, n (%)	55 (68.8)	24 (60)	31 (77.5)	NS
Diabetes, n (%)	20 (25)	10 (25)	10 (25)	NS
Obesity, n (%)	35 (43.8)	16 (40)	19 (47.5)	NS
Chronic kidney disease, n (%)	5 (6.3)	2 (5)	3 (7.5)	NS
Oncopathology, n (%)	3 (3.8)	1 (2.5)	2 (5)	NS
Clinical assessment at baseline				
Loss of smell / taste, n (%)	67 (83.8)	33 (82.5)	34 (85)	NS
Sore throat, n (%)	48 (60)	22 (55)	26 (65)	NS
Dyspnea, n (%)	58 (72.5)	30 (75)	28 (70)	NS
Dry cough, n (%)	68 (85)	36 (90)	32 (80)	NS
Fever, n (%)	75 (93.8)	38(95)	37 (92.5)	NS
Need for oxygen, n (%)	40 (50)	22 (55)	18 (45)	NS
Body temp °C, mean±SD	37.8±0.7	37.6±0.7	38.0±0.7	NS
BP syst., mm Hg, mean±SD	130.6±10.0	130.6±10.5	137.5±22.5	NS
BP diast., mm Hg mean±SD	85.6±10.5	86.0±8.6	85.25±12.40	NS
Respiratory rate/min, mean±SD	24.5±4.4	24.8±4.6	24.2±3.1	NS
CRP, mg/l, mean±SD	69.1±17.3	72.4±16.7	65.8±17.9	NS
D-dimer, ng/ml, mean±SD	746.1±109.4	736.7±112.6	755.5±106.1	NS
Fasting glucose, mmol/l, mean±SD	6.4±1.9	5.9±1.5	6.9±2.2	NS
[K+] plasma, mmol/l	4.29±0.36	4.36±0.32	4.22±0.40	NS

**Table:** General characteristics of Study2.

Characteristics	Overall (n=20)
Sex, females,n (%)	8 (40)
Age, years, mean±SD	44.8±13.9
Hypertension, n (%)	5 (25)
Diabetes, n (%)	2 (10)
Obesity, n (%)	4 (20)

SD, standard deviation; NS, Non-significant difference; mean BP, mean blood pressure; [K+] plasma, plasma concentration of potassium; CRP, C-reactive protein.

Before treatment and after 5 days on either HiIDex (Group I) or SpiDex (Group II) body temperature, cough frequency, blood pressure, respiratory rate, and blood oxygen levels (%) were recorded. C-reactive protein (CRP), D-dimer, and fasting blood glucose were determined at the same time. Chest X-rays were taken prior to treatment and on day 5 after starting treatment. Composite outcomes including clinical improvement, radiological evidence of pneumonia resolution or deterioration, need for invasive ventilatory support and/or all-cause inhospital mortality were noted during in-hospital follow-up.

### Study 2

This was a study of the safety of treating 20 outpatients with PCR-confirmed COVID-19 without pneumonia within 2-3 days of first symptoms. They were treated with dexamethasone 4 mg daily and spironolactone 100 mg daily. The general characteristics, co-morbidities and clinical parameters of these patients are listed in **Table 1**. Based on the results of the large RECOVERY study which showed no benefit of dexamethasone alone in patients with COVID-19 who were not on oxygen therapy or invasive ventilation we did not feel that it was ethical to have a dexamethasone alone control group. The ages of patients ranged from 21 to 58 years.

### Statistical Methods

For normally-distributed demographics and clinical characteristics, data were expressed as the mean and standard deviation. Frequencies and percentages were calculated for categorical variables. The differences between the groups defined as receiving HiDex or SpiDex therapy were compared using Student’s t test. A p value of <0.05 was considered to be statistically significant.

### Safety and Security

All standard biosecurity and institutional safety procedures were adhered to.

## Results

### Study 1

The results of study 1 comparing high dose dexamethasone (HiDex- Group 1) with low dose dexamethasone and spironolactone (SpiDex Group II) are shown in **Table 2**. This details the baseline measurements and those taken after 5 days of treatment. It was not possible to compare the two groups at 10 days as more patients had been discharged from hospital in the SpiDex as compared to the HiDex group. There was 1 death in the HiDex group and no deaths in the SpiDex group. Both groups showed improvement in their clinical parameters but this was more marked in the SpiDex group. Both groups showed a significant reduction in C-Reactive Protein and D-Dimer levels. However, the SpiDex group levels of D-Dimer were significantly lower than those with dexamethasone alone. Not surprisingly both groups had a significant elevation of their fasting blood glucose after 5 days. However, this was significantly lower in the SpiDex group as compared to the HiDex. All patients had radiological evidence of pneumonia at baseline. At 5 days 24 out of 40 patients on HiDex still had radiological changes as compared to 15 on SpiDex. Of note 5 patients in the HiDex group had radiological deterioration at 5 days in comparison to none in the SpiDex group.

Table 2Study 1. Repeated measures analysis comparing changes in variables from baseline to 5 days treatment (T1).

**Table:** 

Dependent variable	Treatment group	Mean baseline	Mean T1	p-value of comparison baseline vs T1	p-value of T1 comparison of Group II vs Group I
Loss of smell/taste, n	I	33 (82.5)	30 (75)	NS	<0.05
	II	34 (85)	19 (47.5)	<0.05	
Dyspnea, n	I	30 (75)	10 (25)	<0.001	NS
	II	28 (70)	8 (20)	<0.001	
Dry cough, n	I	36 (90)	18 (45)	<0.001	NS
	II	32 (80)	12 (30)	<0.001	
Fever, n	I	38 (95)	10 (25)	<0.001	NS
	II	37 (92.5)	4 (10)	<0.001	
Need for oxygen, n	I	22 (55)	7 (17.5)	NS	NS
	II	18 (45)	5 (12.5)	NS	
BP syst., mm Hg	I	130.6±10.5	165.1±12.1	<0.05	<0.05
	II	137.5±22.5	128.3±12.0	NS	
BP diast., mm Hg	I	86.0 ± 8.6	89.0 ± 10.6	NS	NS
	II	85.25 ± 12.4	81.1 ± 6.7	NS	
Breath rate/min	I	24.8 ± 4.6	18.4 ± 3.1	NS	NS
	II	24.2 ± 3.1	16.2 ± 1.8	<0.05	
CRP, mg/l	I	72.4 ± 16.7	28.1 ± 12.9	<0.05	NS
	II	65.8 ± 17.9	17.1 ± 8.7	<0.05	
D-dimer, ng/ml	I	736.7 ± 112.6	574.8 ± 106.4	NS	<0.05
	II	755.5 ± 106.1	357.3 ± 71.1	<0.001	
Fasting glucose, mmol/l	I	5.9 ± 1.5	12.69 ± 1.31	<0.05	<0.05
	II	6.9 ± 2.2	8.44 ± 1.60	NS	
[K+] plasma, mmol/l	I	4.36 ± 0.32	4.28 ± 0.50	NS	NS
	II	4.22 ± 0.40	4.43 ± 0.45	NS	

SD, standard deviation; mean BP, mean blood pressure; [K+] plasma, plasmaconcentration of potassium; CRP, C-reactive protein;Figures are expressed as Mean and Standard DeviationNS Non-significant differenceNo patient required ventilation: patients with oxygen saturation less than 92% given oxygen by mask.

**Table:** Study 2. Repeated measures analysis comparing changes in variables from baseline to 5 days treatment (T1) and 10 days treatment (T2).

Dependent variable	Mean baseline	Mean T1	p-value of comparison baseline vs T1	Mean T2	p-value of comparison baseline vs T2
Loss of smell / taste, n (%)	18 (90)	8 (40)	<0.005	1 (5%)	<0.001
Sore throat, n (%)	11 (55)	3(15)	<0.05	0	<0.001
Dyspnea, n (%)	10 (50)	6 (30)	<0.05	2 (10)	<0.05
Dry cough, n (%)	16 (80)	11 (55)	<0.05	2 (10)	<0.001
Fever, n (%)	16 (80)	4 (20)	<0.005	0	<0.001
PCR COVID-19	20 (100)	6 (30)	<0.001	2(10)	<0.001
positive, n (%)					
Blood oxygen, % mean±SD,	96.5±1.6	97.7±1.9	NS	98.6±1.4	NS
Body temp °C, mean±SD	37.8±0.7	36.8±0.2	<0,05	36.6±0.2	<0.05
[K+] plasma, mmol/l	4.29±0.36	4.36±0.32	NS	4.22±0.20	NS

SD, standard deviation; [K+] plasma, plasma concentration of potassium; PCR polymerase chain reaction test.

### Study 2

The results of the outpatient study designed to test the safety of the regimen are given in **Table 2**. The classical symptoms and signs of COVID-19 were markedly improved after 5 days and at 10 days were almost completely resolved. Thus, only 1 patient had loss of smell and taste at 10 days as compared to 18 out of 20 at baseline. 10% had breathlessness at 10 days compared to 50% at baseline. The dry cough was a presenting feature in 80% patients but had resolved in all but 10% by 10 days. No patient became hyperkalaemic and no adverse effects were reported. There was a need for hospitalisation for 2 patients during the 10-day period of our outpatient observation. Both admissions were for co-morbidities: one was for a transient ischaemic attack and the other for a urinary tract infectio)n in a patient with type II diabetes. There were no COVID-associated complications during SpiDex treatment. For reasons discussed above we did not feel that it was ethical to have a HiDex outpatient control group. However, without an untreated control group we cannot be sure what, if any, benefit ensues from this treatment.

## Discussion

This paper tries to explain the consequences of the SARS-CoV-2 virus infecting key cells which express ACE2 receptors, mineralocorticoid receptors and the cortisol inactivating enzyme 11βHSD2. These cells are normally aldosterone selective but SARS-CoV-2 infection results in a loss of the specificity of the mineralocorticoid receptor protective mechanism. It is well known that the MR can be activated by normal levels of glucocorticoids when tissues are damaged by Reactive Oxygen Species generation (12, 34). SARS-CoV-2 by destroying the ACE2 receptor produces high levels of angiotensin II which stimulate NADPH oxidase and hence ROS production. In addition it has been shown that aldosterone induces oxidative stress in both aortic tissue and macrophages and that this can be blocked by MR antagonists (35)

This loss of MR selectivity allows cortisol to activate the MR and stimulate the basolateral release of ATP. The subsequent increase in intracellular calcium following purinergic receptor activation then stimulates the exocytosis of the ATP containing lysosomes. This is similar to the process that has been described for the exocytosis of Weibel-Palade bodies from endothelial cells (18, 27). It is suggested that the high level of extracellular ATP then acts on adjacent cells to produce loss of smell, taste and a non-productive cough. This would explain why the duration of the anosmia and ageusia in COVID-19 patients is much shorter than that seen with other viruses with cell destruction. If this is true it is likely that recovery relates to the decreased lysosomal exocytosis of ATP.

The two proof of concept studies produce important evidence that favours the hypothesis. In the first inpatient study we compared high dose dexamethasone with low dose dexamethasone and spironolactone. In the outpatient study we used low dose dexamethasone and spironolactone and had no control group given dexamethasone alone. This was on the basis of the observations made in the RECOVERY study which showed that patients with COVID-19 who were not on oxygen did not benefit from dexamethasone (36).

In the RECOVERY trial it was thought that high dose dexamethasone might have produced benefit by reducing inflammatory-based lung injury (37). If this was the main mechanism of action it might be expected that high dose dexamethasone (16 mg/day) would have a significantly greater effect than low dose dexamethasone (4 mg/day) and spironolactone. The results of the study listed in **Table 2** do not support this. Systemic inflammation as measured by C-reactive Protein is strongly associated with severity and mortality in COVID-19. We found that CRP levels were lowered by high dose dexamethasone (HiDex) but that low dose dexamethasone and spironolactone (SpiDex) produced a further reduction of CRP levels. D-dimer levels were not significantly reduced by HiDex but were highly significantly reduced by SpiDex. Not surprisingly the SpiDex D-dimer results were significantly lower than HiDex. This is important given that D-dimer levels are a key measure of thrombotic activity. Patients with high D-dimer and high CRP have the greatest risk of adverse outcomes with COVID-19 (38). Part of the beneficial anti-inflammatory effect of the combination of low dose dexamethasone and spironolactone could be the result of blocking the release of the NLRP3 inflammasome *via* lowering extracellular ATP levels. Freeman and Swartz (25) have identified this inflammasome as a key target for the treatment of severe COVID-19 infection.

If, as suggested, the non-productive cough and loss of taste and smell, the characteristic symptoms of COVID-19, are due to purinergic activation following the exocytosis of ATP then it might be thought that lowering ATP levels would have a beneficial effect. This appeared to be so. In the inpatient study cough was present in 90% of the HiDex group at baseline and in 45% at 5 days. In the SpiDex group the baseline was 89% and 30% at 5 days. The recovery of taste and smell was more striking. At baseline 82.5% of the HiDex group had anosmia/ageusia. This fell to 75% at 5 days. In the SpiDex group this was 85% at baseline and 47.5% at 5 days (significantly lower than the HiDex group). In the outpatient study 90% of the patients had loss of taste and smell at baseline but only 1 patient (5%) had this symptom after 10 days of SpiDex treatment.

**Figure 3:**
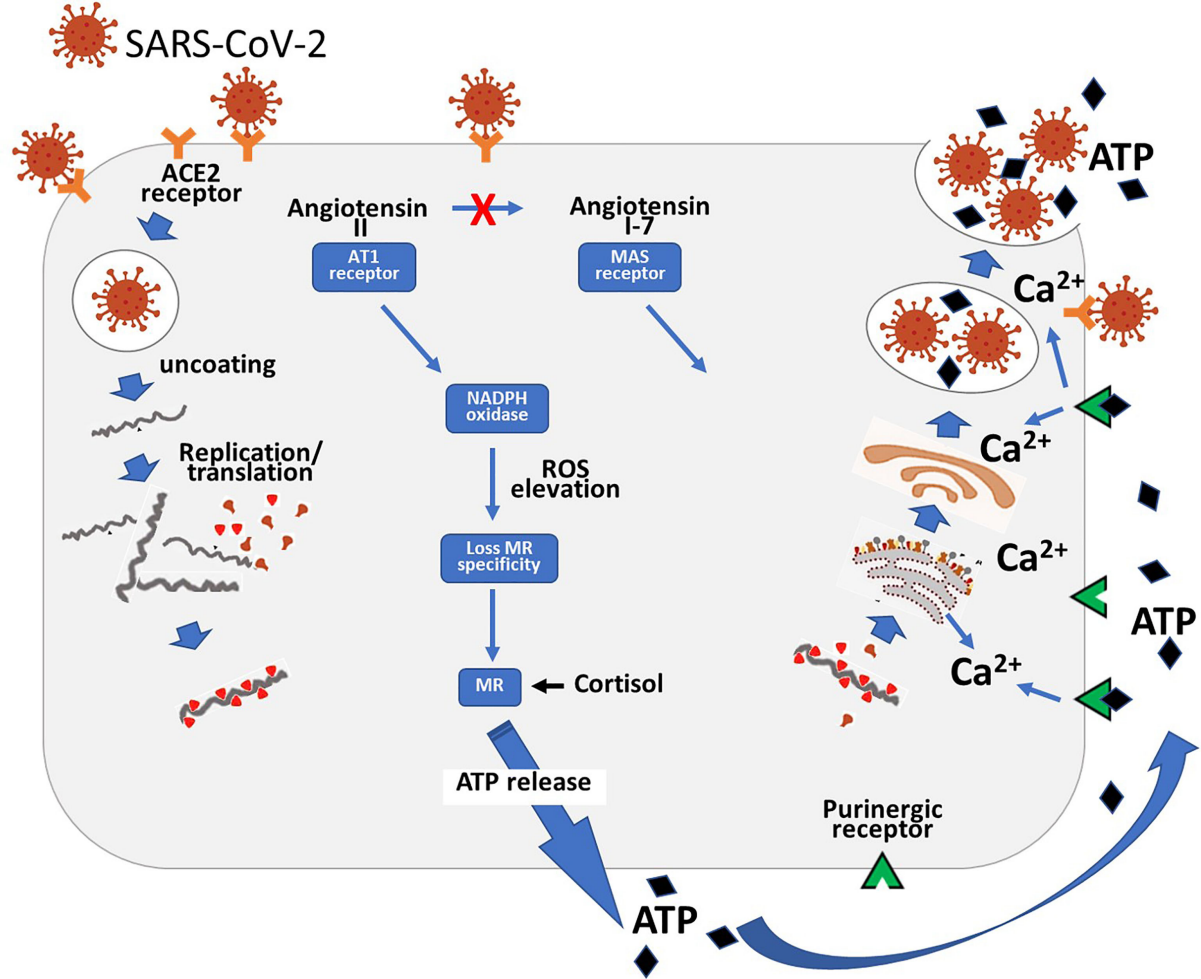
Diagram showing the entry of the SARS-CoV-2 virus into the cell *via* the ACE2 receptor. Following replication of the virus it hijacks lysosomes. The high level of angiotensin II consequent on the loss of the ACE2 receptor results in ROS production with consequent loss of MR specificity. The MR is then stimulated by cortisol which stimulates basolateral release of ATP. This then acts *via* adjacent purinergic receptors to increase intracellular calcium. This then produces exocytosis of the ATP and viral containing lysosomes.

The mechanism by which the SARS-CoV-2 virus gets into cells using the ACE2 receptor has been extensively studied. Much less well understood is how the virus exits the cell. An important paper by Ghosh et al. (39) has suggested that the virus gets out of the cell not by cell lysis or the normal bio-secretory pathway but by hijacking lysosomes (**Figure 3**). In this process the normally acidic lysosomes are de-acidified. This change in pH markedly stimulates this exocytic pathway. The reason for the change in pH is not clear but studies have shown that angiotensin II regulates the Sodium/Hydrogen Exchanger (40). The loss of protons from the cell in exchange for sodium raises intracellular pH. The NHE proteins 1-5 are on the cell surface. Intracellularly there is a subfamily of NHE 6-9 that are localised to the membranes of organelles including late endosomes which fuse with lysosomes. Studies have shown that lysosomal deacidification is key for the opening of the pH dependent calcium channel in the wall of the lysosome (41). This channel, TRPML3, is only active at neutral pH. Entry of calcium into the lysosome stimulates exocytosis.

As discussed above ATP can be released from the cell by lysosomal exocytosis. This raises a fascinating question – could inhibition of the release of lysosomes have an effect on both ATP and SARS-CoV-2 exocytosis? The evidence reviewed above demonstrates that MR blockade can inhibit the release of ATP. The results obtained by Lin et al. (33) showed that the addition of spironolactone to various combinations of anti-viral drugs significantly reduced the time period of viral detection in nasal swabs. Unfortunately, they did not test spironolactone alone.

In addition to its conventional diuretic effect and its inhibition of exocytosis it is of interest that MR receptor antagonism with spironolactone has been shown to markedly increase the synthesis and expression of ACE2 receptors (42). If the mechanism described in this paper is true, then the duration of loss of the ACE2 receptors in specific cell types could be important. This possibility and its potential treatment with spironolactone need to be tested in the context of Long COVID.

If this hypothesis is correct then following your nose may provide an important clue as to how the SARS-CoV-2 works and also provide insight into how this very challenging condition can be treated.

## Data Availability Statement

The original contributions presented in the study are included in the article/supplementary material, further inquiries can be directed to the corresponding author.

## Ethics Statement

The studies involving human participants were reviewed and approved by the University Bioethical Committee, Vinnytsia National Pirogov Memorial Medical University, Vinnytsia, Ukraine. The patients/participants provided their written informed consent to participate in this study.

## Author Contributions

CE - hypothesis, original clinical studies on specificity of MR, purinergic receptors, and ATP release. OK- clinical studies in Ukraine. LH - clinical studies in Ukraine. YK- laboratory studies using scanning ion conductance microscopy to study action of aldosterone. All authors contributed to the article and approved the submitted version.

## Conflict of Interest

The authors declare that the research was conducted in the absence of any commercial or financial relationships that could be construed as a potential conflict of interest.

## Publisher’s Note

All claims expressed in this article are solely those of the authors and do not necessarily represent those of their affiliated organizations, or those of the publisher, the editors and the reviewers. Any product that may be evaluated in this article, or claim that may be made by its manufacturer, is not guaranteed or endorsed by the publisher.
