# Erratum: Follow Your Nose: A Key Clue to Understanding and Treating COVID-19

**DOI:** 10.3389/fendo.2021.830164

**Published:** 2021-12-16

**Authors:** Frontiers Production Office

**Affiliations:** Frontiers Media SA, Lausanne, Switzerland

**Keywords:** COVID-19, mineralocorticoid receptor, spironolactone and dexamethasone, ATP - adenosine triphosphate, anosmia and ageusia

Owing to a production error, several dependent variables were omitted from the part of **Table 2** that pertains to Study 1 in the article as originally published. The variables in question are mean diastolic blood pressure, breath rate, C-reactive protein concentration, D-dimer concentration, fasting glucose concentration and plasma potassium concentration. The complete part of **Table 2** that pertains to Study 1 is presented here.

**Table 2:** Study 1. Repeated measures analysis comparing changes in variables from baseline to 5 days treatment (T1).

Dependent variable	Treatment group	Mean baseline	Mean T1	p-value of comparison baseline vs T1	p-value of T1 comparison of Group II vs Group I
Loss of smell/taste, n	I	33 (82.5)	30 (75)	NS	<0.05
	II	34 (85)	19 (47.5)	<0.05	
Dyspnea, n	I	30 (75)	10 (25)	<0.001	NS
	II	28 (70)	8 (20)	<0.001	
Dry cough, n	I	36 (90)	18 (45)	<0.001	NS
	II	32 (80)	12 (30)	<0.001	
Fever, n	I	38 (95)	10 (25)	<0.001	NS
	II	37 (92.5)	4 (10)	<0.001	
Need for oxygen, n	I	22 (55)	7 (17.5)	NS	NS
	II	18 (45)	5 (12.5)	NS	
BP syst., mm Hg	I	130.6 ± 10.5	165.1 ± 12.1	<0.05	<0.05
	II	137.5 ± 22.5	128.3 ± 12.0	NS	
BP diast., mm Hg	I	86.0 ± 8.6	89.0 ± 10.6	NS	NS
	II	85.25 ± 12.4	81.1 ± 6.7	NS	
Breath rate/min	I	24.8 ± 4.6	18.4 ± 3.1	NS	NS
	II	24.2 ± 3.1	16.2 ± 1.8	<0.05	
CRP, mg/l	I	72.4 ± 16.7	28.1 ± 12.9	<0.05	NS
	II	65.8 ± 17.9	17.1 ± 8.7	<0.05	
D-dimer, ng/ml	I	736.7 ± 112.6	574.8 ± 106.4	NS	<0.05
	II	755.5 ± 106.1	357.3 ± 71.1	<0.001	
Fasting glucose, mmol/l	I	5.9 ± 1.5	12.69 ± 1.31	<0.05	<0.05
	II	6.9 ± 2.2	8.44 ± 1.60	NS	
[K+] plasma, mmol/l	I	4.36 ± 0.32	4.28 ± 0.50	NS	NS
	II	4.22 ± 0.40	4.43 ± 0.45	NS	

SD, standard deviation; mean BP, mean blood pressure; [K+] plasma, plasma concentration of potassium; CRP, C-reactive protein. Figures are expressed as Mean and Standard Deviation. NS, non-significant difference. No patient required ventilation: patients with oxygen saturation less than 92% given oxygen by mask.

The publisher apologizes for this error. The original article has been updated.

